# Distinct Spatiotemporal Dynamics of Peptidoglycan Synthesis between *Mycobacterium smegmatis* and *Mycobacterium tuberculosis*

**DOI:** 10.1128/mBio.01183-17

**Published:** 2017-09-12

**Authors:** Helene Botella, Guangli Yang, Ouathek Ouerfelli, Sabine Ehrt, Carl F. Nathan, Julien Vaubourgeix

**Affiliations:** a Department of Microbiology and Immunology, Weill Cornell Medical College, New York, New York, USA; b Organic Synthesis Core Facility, Chemical Biology Program, Memorial Sloan Kettering Cancer Center, New York, New York, USA; Washington University in St. Louis School of Medicine

**Keywords:** cell division, infectious diseases, microbiology, mycobacteria, peptidoglycan, tuberculosis

## Abstract

Peptidoglycan (PG), a polymer cross-linked by d-amino acid-containing peptides, is an essential component of the bacterial cell wall. We found that a fluorescent d-alanine analog (FDAA) incorporates chiefly at one of the two poles in *Mycobacterium smegmatis* but that polar dominance varies as a function of the cell cycle in *Mycobacterium tuberculosis*: immediately after cytokinesis, FDAAs are incorporated chiefly at one of the two poles, but just before cytokinesis, FDAAs are incorporated comparably at both. These observations suggest that mycobacterial PG-synthesizing enzymes are localized in functional compartments at the poles and septum and that the capacity for PG synthesis matures at the new pole in *M. tuberculosis*. Deeper knowledge of the biology of mycobacterial PG synthesis may help in discovering drugs that disable previously unappreciated steps in the process.

## IMPORTANCE

People are dying all over the world because of the rise of antimicrobial resistance to medicines that could previously treat bacterial infections, including tuberculosis. Here, we used fluorescent d-alanine analogs (FDAAs) that incorporate into peptidoglycan (PG)—the synthesis of which is an attractive drug target—combined with high- and super-resolution microscopy to investigate the spatiotemporal dynamics of PG synthesis in *M. smegmatis* and *M. tuberculosis*. FDAA incorporation predominates at one of the two poles in *M. smegmatis*. In contrast, while FDAA incorporation into *M. tuberculosis* is also polar, there are striking variations in polar dominance as a function of the cell cycle. This suggests that enzymes involved in PG synthesis are localized in functional compartments in mycobacteria and that *M. tuberculosis* possesses a mechanism for maturation of the capacity for PG synthesis at the new pole. This may help in discovering drugs that cripple previously unappreciated steps in the process.

## INTRODUCTION

Rod-shaped bacteria like *Escherichia coli* and *Bacillus subtilis* elongate by adding new peptidoglycan (PG) along the lateral body (1–4). This so-called dispersed cell growth contrasts with zonal cell growth, which restricts the addition of cell building blocks to specific locations, including the cell poles. Polar growth prevails in Gram-negative *Rhizobiales* and in Gram-positive *Actinomycetales*, which include mycobacterial species (5–11). The selective advantage of polar growth over dispersed growth in certain species remains unknown, but it has been suggested that such growth may foster cell-to-cell heterogeneity and favor rejuvenation of progeny by selectively passing on intact proteins and cell wall components to newborn cells (8, 12–14).

**FIG 1:**
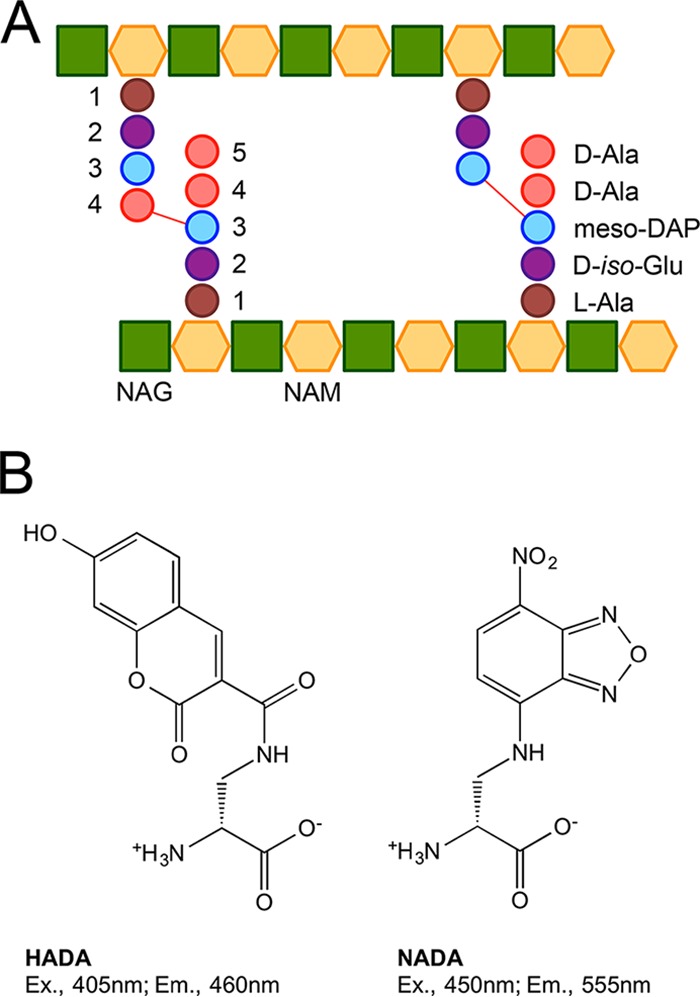
Composition of *M. tuberculosis* peptidoglycan and structures of the fluorescent d-alanine analogs that allow its visualization. (A) Schematic representation of *M. tuberculosis* peptidoglycan (PG). *M. tuberculosis*’s PG is composed of strands of a repeating unit of the disaccharide *N*-acetyl-glucosamine (NAG) and *N*-acetyl-muramic acid (NAM). The strands are linked by peptide chains composed of l-alanine (l-Ala), d-*iso*-glutamine (d-*iso*-Glu, a product of amidation of d-Glu), *meso*-diaminopimelic acid (meso-DAP), and d-alanine (d-Ala). (B) Chemical structures of the fluorescent d-alanine analogs, HADA and NADA. Ex., excitation; Em., emission.

PG is a rigid exoskeleton composed of strands of repeating units of the disaccharide *N*-acetyl-glucosamine (NAG)–*N*-acetyl-muramic acid (NAM) cross-linked by peptide chains (Fig. 1A). Penicillin and other β-lactams, such as carbapenems and cephalosporins, which commonly target the bacterial cell wall by halting peptidoglycan biosynthesis in replicating bacteria, are the antibiotics most widely used to treat Gram-positive and Gram-negative bacterial infections. β-Lactams often cause bacterial lysis (15, 16). Disruption of the balance between PG synthesis and PG rupture by hydrolases generates a futile cycle that depletes cellular resources, contributing to cell death (17).

Penicillin and other β-lactams were long considered ineffective for the treatment of tuberculosis (TB) because *Mycobacterium tuberculosis* encodes the class A (Ambler) β-lactamase BlaC that hydrolyzes many β-lactams, destroying their bactericidal activity (18). However, recent studies have prompted reconsideration of the use of β-lactams for TB therapy (19–23).

Despite the importance in medicine of antibiotics that target PG, the lack of adequate tools hindered deeper investigation and improved comprehension of PG dynamics, especially in live organisms, until the development of fluorescent d-alanine analogs (FDAA) (9, 24–31). Two routes ensure the incorporation of FDAAs into bacterial PG: periplasmic editing of mature PG by l,d-transpeptidases or d,d-transpeptidases and cytosolic incorporation into PG precursors by intracellular ligases. Using chemical reporters of growth—either an FDAA or an amine-reactive dye—it was reported that *Mycobacterium smegmatis* elongates preferentially from the old pole (6–9), although this was disputed (32). However, the mode of elongation of wild-type (WT) *M. tuberculosis* remained largely unexplored.

Here, we used FDAA (Fig. 1B) to delineate fundamental differences in the modes of elongation of *M. smegmatis* and *M. tuberculosis*. A better definition of the repertoire of the synthetic machineries that incorporate PG into mycobacteria will help rationalize the modes of action of the growing number of β-lactam antimicrobials under consideration for use in TB therapy.

## RESULTS AND DISCUSSION

**FIG 2:**
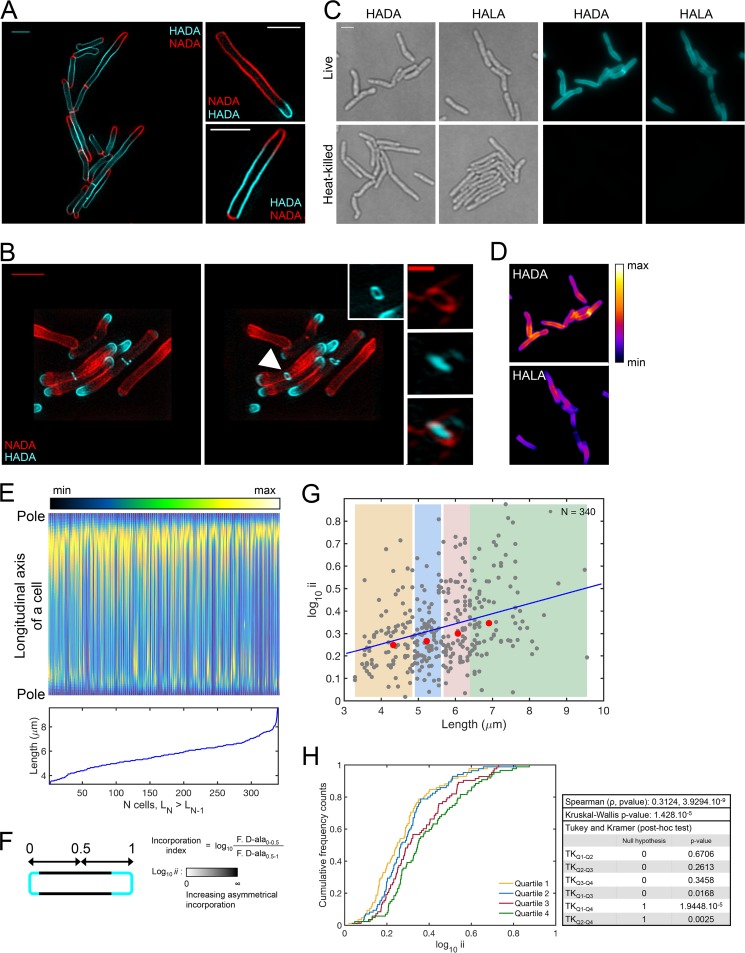
*M. smegmatis* elongates chiefly from one of the two poles. (A) Examples of super-resolution photographs of *M. smegmatis* cells labeled sequentially with HADA for 4 h and then with NADA for 30 min or labeled sequentially with NADA for 4 h and then with HADA for 30 min. Scale bar, 2 μm. Short-pulsed d-alanine analogs incorporated asymmetrically at the two poles. (B) Super-resolution photographs of *M. smegmatis* labeled sequentially with NADA for 4 h and HADA for 30 min. The middle panel shows the same cells as in the left panel after a 36° rotation around the *y* axis. Arrowhead, open-ring septum. Inset, enlarged photograph of an open-ring septum. Scale bar, 2 μm. The right panel shows an example of a septum that was labeled with both NADA and HADA. Scale bar, 0.5 μm. (C) Labeling of live or heat-killed *M. smegmatis* cells with 1 mM HADA and 1 mM HALA. Grey, transmitted-light snapshots. Scale bar, 2 μm. (D) False-colored fluorescence intensities of cells in snapshots presented in panel C. (E) (Top) Fluorescence intensities along the longitudinal axes of 340 cells labeled with 1 mM HADA for 30 min and sorted from the shortest (left) to the longest (right). (Bottom) Absolute lengths of the 340 cells sorted from the shortest (left) to the longest (right). (F) The incorporation index (log_10_ ii) is the logarithm of the ratio of the fluorescence intensities of the 2 halves of a cell. For short-pulsed cells, the log_10_ ii documents whether the incorporation of d-alanine analogs at the poles is symmetrical (log_10_ ii of ~0) or asymmetrical (log_10_ ii of >0). (G) Log_10_ ii values of all 340 cells in panel E relative to their lengths. Each dot represents a single cell profiled from 4 independent experiments. The yellow, blue, red, and green shaded areas represent the first, second, third, and fourth size quartiles, respectively. The red dots represent the median values for size and the log_10_ ii values of all cells in each quartile. The blue line indicates the Spearman correlation. Cells bearing a septum were excluded from the analysis. (H) Cumulative distribution function of the incorporation index. For each quartile color coded in panel G, the curve reports the cumulative frequency cell counts as a function of the log_10_ ii. This indicates the distribution of the log_10_ ii values in each quartile and allows comparisons among them. The table includes the following: the Spearman correlation coefficient; its associated *P* value; the Kruskal-Wallis multiple-comparison test (a significant Kruskal-Wallis test indicates that a minimum of one quartile differs from the others); and the *post hoc* two-by-two comparison of each quartile with another using a Tukey-Kramer procedure. For the latter statistical test, the null hypothesis is that the data in two quartiles are from the same continuous distribution (value of 0); the alternative hypothesis is that the data in two quartiles are from different continuous distributions (value of 1). The result is 1 if the test rejects the null hypothesis at the 1% significance level and 0 otherwise.

First, we evaluated how FDAAs incorporate into growing *M. smegmatis* cells. *M. smegmatis* cells were perfused with 7H9 medium supplemented with 1 mM 4-chloro-7-nitrobenzofurazan (NBD-Cl) coupled to 3-amino-d-alanine (NADA) (25). After 4 h, the cells were collected, washed, and transferred for 30 min into fresh 7H9 medium supplemented with 1 mM 7-hydroxycoumarin-3-carboxylic acid (HCC-OH) coupled to 3-amino-d-alanine (HADA). Neither NADA nor HADA at this concentration impairs cell growth (31). Inspection by super-resolution microscopy of labeled *M. smegmatis* cells revealed that the incorporation of FDAAs occurred predominantly at one pole (Fig. 2A). Three-dimensional structured-illumination microscopy (3-D–SIM) allowed us to monitor the progress of cytokinesis (Fig. 2B). Septum formation progressed through concentric incorporation of PG, from outer to inner layers (Fig. 2B). Heat-killed *M. smegmatis* cells failed to incorporate FDAAs, suggesting that the fluorescence observed after perfusion with FDAAs is not attributable to nonspecific binding to the mycobacterial cell wall (Fig. 2C). In contrast to what has been reported in *E. coli*, *Agrobacterium tumefaciens*, and *B. subtilis* (25), fluorescent l-alanine analogs (FLAAs) stained the periphery of *M. smegmatis* cells, although their fluorescence intensity was only half that afforded by FDAAs (Fig. 2C and D). Next, we measured the fluorescence intensities of single cells along the longitudinal axis (Fig. 2E) and computed an incorporation index (log_10_ ii), the logarithm of the ratio of the fluorescence intensities of a cell’s two halves (Fig. 2F and G). The median value of this index was 0.29. Greater incorporation of FDAAs at one pole suggests that *M. smegmatis* elongates asymmetrically from a preferred pole (Fig. 2A, E, G, and H), as reported previously (6–9). Longer *M. smegmatis* cells had greater incorporation indices (Fig. 2G and H). This is consistent with the finding that the growth rate of an *M. smegmatis* cell correlated positively with its size (33).

**FIG 3:**
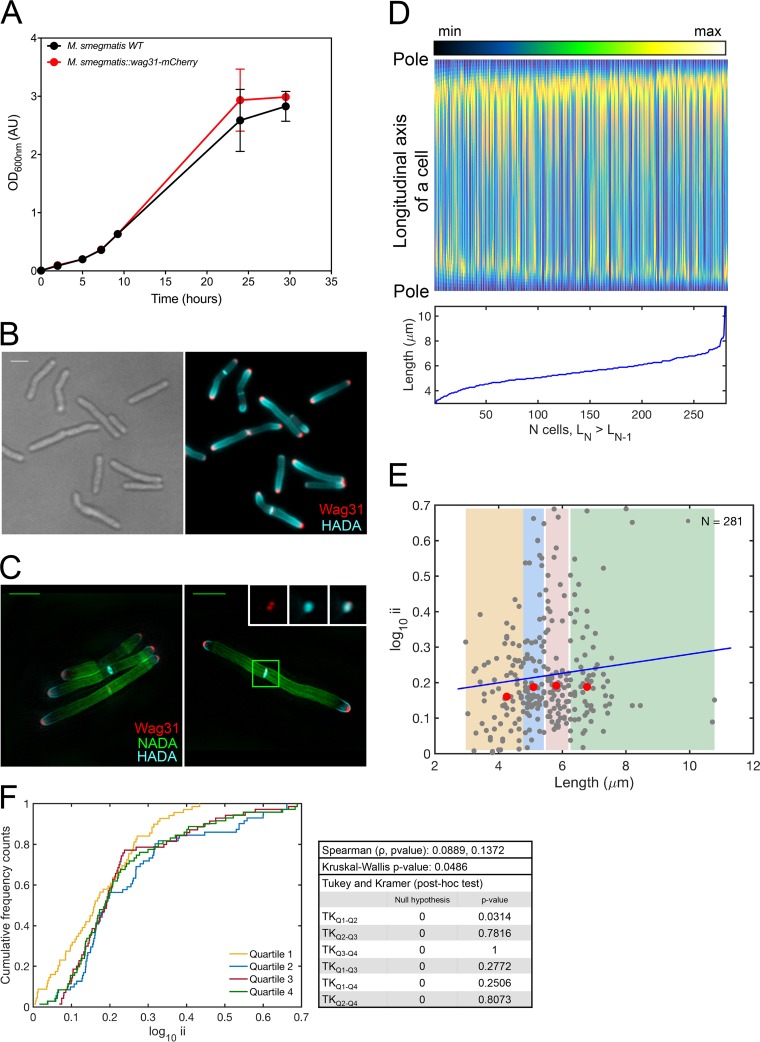
Expression of Wag31-mCherry affects *M. smegmatis* cells’ elongation. (A) Merodiploid expression of Wag31-mCherry does not alter the growth of *M. smegmatis*. (B) *M. smegmatis* Wag31-mCherry cells perfused for 30 min with 1 mM HADA. Scale bar, 2 μm. (C) Super-resolution photographs of *M. smegmatis* Wag31-mCherry cells labeled sequentially with NADA for 4 h and HADA for 30 min. (Inset) Enlarged photograph, after a 48° rotation around the *y* axis, of the closed-ring septum boxed in green. Two Wag31-mCherry foci localized to the septum. Scale bar, 2 μm. (D) (Top) Fluorescence intensities along the longitudinal axes of 281 cells, sorted from the shortest (left) to the longest (right). (Bottom) Absolute lengths of the 281 cells, sorted from the shortest (left) to the longest (right). (E) Log_10_ ii values of all 281 cells in panel D relative to their lengths. The 281 cells profiled are from 3 independent experiments. The yellow, blue, red, and green shaded areas represent the first, second, third, and fourth size quartiles, respectively. The red dots represent the median values for size and the log_10_ ii values of all cells in each quartile. The blue line indicates the Spearman correlation. Cells bearing a septum were excluded from the analysis. (F) Cumulative distribution functions of the incorporation index. For each quartile color coded in panel E, the curve reports the cumulative frequency cell count as a function of the log_10_ ii. This indicates the distribution of the log_10_ ii values in each quartile and allows comparisons among them. Statistical analysis is as described in the legend to Fig. 2H.

Rather than cytoskeleton-like proteins, *Actinobacteria* use the coiled-coil protein DivIVA to guide cell wall synthesis spatially. The protein Wag31—the DivIVA homolog in mycobacteria—recognizes membrane curvature to ensure its localization to the poles (9, 34). It is likely that Wag31 anchors the incipient elongation complex at the pole and serves as a structural basis for the recruitment of the remaining components of the complex after it has been phosphorylated and stabilized by CwsA (11, 35–37). In mycobacteria, Wag31 tagged with a fluorescent reporter has been extensively used to track cell division (9, 32, 38). Thus, we evaluated the incorporation of FDAAs in *M. smegmatis* cells that expressed an additional copy of Wag31. We transformed WT *M. smegmatis* with a plasmid that encodes Wag31-mCherry. The *M. smegmatis* Wag31-mCherry strain grew similarly to WT *M. smegmatis* (Fig. 3A). When visualized by high-resolution microscopy, Wag31-mCherry appeared as a single focus at the poles and a single focus at the septum until the onset of separation of a progeny pair, as reported previously (32) (Fig. 3B). However, 3-D–SIM inspection revealed two distinct foci, located at each side of the septal plane (Fig. 3C). In contrast to WT *M. smegmatis*, the levels of incorporation of FDAAs at the two poles were more comparable (Fig. 3B to F), giving a median value of 0.18 for the incorporation index.

Thus, the expression of an additional tagged copy of Wag31 expressed under a strong mycobacterial promoter in *M. smegmatis* perturbed the normal pattern of FDAA incorporation in growing cells, which elongated more similarly at both poles (Fig. 3E and F). Similarly, Meniche and collaborators documented that the expression of an additional copy of Wag31 from a strong promoter led to a relatively even distribution of Wag31 at the two poles, although it was still preferentially concentrated at the old pole (9).

**FIG 4:**
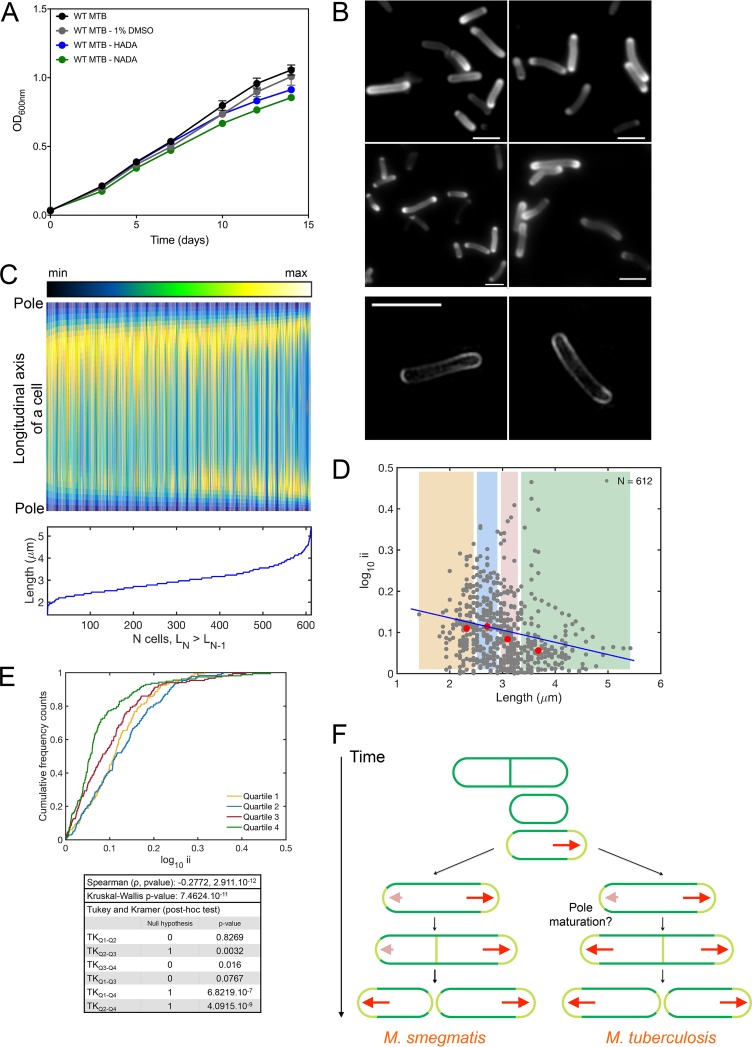
Variations in polar dominance of FDAA incorporation as a function of cell cycle in *M. tuberculosis*. (A) *M. tuberculosis* growth is not impaired for up to 7 days of perfusion with 1 mM HADA or 1 mM NADA. The final concentration of DMSO in 7H9 medium supplemented with d-alanine analogs was 1%. (B) (Top) *M. tuberculosis* cells perfused for 4 h with 1 mM HADA. Similar levels of cell-to-cell FDAA incorporation were seen with longer perfusion of FDAAs. (Bottom) Examples of super-resolution photographs of *M. tuberculosis* cells labeled with 1 mM HADA for 16 h. Scale bar, 2 μm. (C) (Top) Fluorescence intensities along the longitudinal axes of 612 cells labeled with 1 mM HADA for 4 h, sorted from the shortest (left) to the longest (right). (Bottom) Absolute lengths of the 612 cells, sorted from the shortest (left) to the longest (right). (D) Log_10_ ii values of all 612 cells in panel C relative to their lengths. Each dot represents a single cell profiled from 3 independent experiments. The yellow, blue, red, and green shaded areas represent the first, second, third, and fourth size quartiles, respectively. The red dots represent the median values for size and the log_10_ ii values for all cells in each quartile. The blue line indicates the Spearman correlation. Cells bearing a septum were excluded from the analysis. (E) Cumulative distribution functions of the incorporation index. For each quartile color coded in panel D, the curve reports the cumulative frequency cell count as a function of the log_10_ ii. This indicates the distribution of the log_10_ ii values in each quartile and allows comparisons among them. Statistical analysis is as described in the legend to Fig. 2H. (F) Schematic representation of the modes of elongation of *M. smegmatis* and *M. tuberculosis* cells.

The results with *M. smegmatis* were not predictive of what we observed with *M. tuberculosis*. The addition of 1 mM FDAAs to 7H9 medium for up to 7 days did not affect the growth of *M. tuberculosis* (Fig. 4A). *M. tuberculosis* cells were perfused with 7H9 medium supplemented with 1 mM HADA or 1 mM NADA for 4 h. Overall, cell-to-cell incorporation of FDAAs was more heterogeneous in *M. tuberculosis* than in *M. smegmatis*. This was a striking difference between the two species. The median value of the incorporation index was 0.09. In contrast to *M. smegmatis*, *M. tuberculosis* incorporated FDAAs heterogeneously: predominantly at one pole in some cells and at both poles in others (Fig. 4B and C). Also in contrast to *M. smegmatis*, the incorporation index of HADA tended to be smaller in longer *M. tuberculosis* cells (Fig. 4C to E). This was indicative of variations in polar dominance as a function of cell cycle.

The quantification of FDAA incorporation into growing cells leads us to propose that *M. smegmatis* and *M. tuberculosis* have distinct modes of elongation (Fig. 4F). FDAA incorporation dominates at one pole in elongating *M. smegmatis* cells independently of their length. This is consistent with numerous reports that *M. smegmatis* elongates preferentially from the old pole (7, 8, 10, 39). In contrast, FDAA incorporation into *M. tuberculosis* varies in polar dominance as a function of the cell’s length. Considering that cell length increases from birth to separation into a progeny pair, we propose that FDAAs are incorporated chiefly at one pole immediately after cytokinesis but comparably at both poles just before cytokinesis (Fig. 4F). These differences likely reflect variations in PG synthetic pathways between the two species. Differences have been reported in the two species’ penicillin-binding proteins (PBPs). *M. smegmatis* encodes three bifunctional PBPs, PonA1 to PonA3, whereas *M. tuberculosis* encodes only PonA1 and PonA2 (40). *M. smegmatis* requires PonA1 for *in vitro* growth, while the PonA1 homolog is dispensable in *M. tuberculosis* (41, 42). However, such differences by themselves do not provide an explanation for our finding that in *M. tuberculosis*, the pole that incorporates FDAAs at a lower rate in a progeny cell at birth matures over the course of the cell cycle to become a pole from which cells elongate at a maximal rate under optimal conditions. The molecular mechanisms by which a cell pole matures remain poorly understood, although some studies have provided some routes of investigation. First, Meniche and colleagues documented that Wag31 is preferentially concentrated at the old pole in *M. smegmatis* when it is expressed at a native level (9). Thus, Wag31 may increasingly accumulate at the incipient pole to help recruit and anchor the elongation complex. Mycobacteria may deploy mechanisms to control the sequestration of Wag31 at the old pole or exclude Wag31 from the new pole. Second, the activity and/or the control of the location of the elongation machinery may be dictated by posttranslational modifications. In this vein, phosphorylation of the peptidoglycan synthase PonA1 governs the rate of polar elongation in *M. smegmatis* (43). Third, Rego and colleagues recently reported that LamA, a member of the mycobacterial division complex, inhibits growth at the incipient pole, thus contributing to asymmetric polar growth (44). Their work suggests that treatment of *M. tuberculosis* with rifampin in combination with an inhibitor of LamA might lead to faster mycobacterial killing. Finally, Eskandarian and colleagues showed that *M. smegmatis* cells present cell surface irregularities, so-called wave troughs (45). *M. smegmatis* progeny cells inherit a wave trough located near the old pole from their mother or grandmother at birth. Asymmetric elongation results in the repositioning of the wave trough to the midcell. Once the wave trough is at midcell, the FtsZ ring forms; septum formation begins 30 min later, cytokinesis is completed after 20 min, and separation of the progeny pair occurs 40 min later. The authors proposed a model in which the division site is placed at a wave trough of the cell and the replicated chromosome negatively regulates the placement of the division site, leaving the midcell the preferred site for division by a mechanism akin to nucleoid occlusion (45). It is possible that the spatiotemporal regulation of the aforementioned mechanisms differs between the two species.

The combination of super-resolution imaging techniques and specific biological probes also has the potential to help increase the knowledge of the modes of action of antimycobacterial compounds by allowing the comparison of morphometric parameters from cells treated with antimycobacterial compounds with unknown modes of action to those of cells treated with compounds with known modes of action (46). Additionally, such studies on drug-treated cells or mutants may help us better understand the coordination of molecular events during cell elongation and cell division.

**FIG 5:**
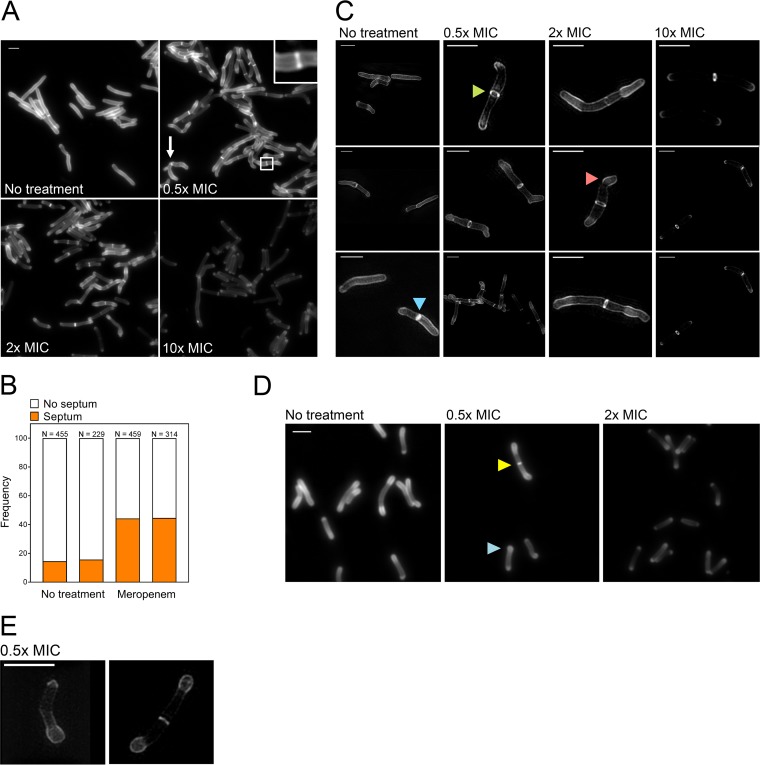
Morphotypes of *M. smegmatis* and *M. tuberculosis* cells treated with meropenem. (A) High-resolution photographs of *M. smegmatis* cells untreated or treated with 0.5×, 2×, and 10× MIC of meropenem for 4 h. Scale bar, 2 μm. Arrow, a bacterium that is undergoing branching. (Inset) Two foci, indicative of an open septum. (B) Proportion of untreated or meropenem-treated (0.5 × MIC) *M. smegmatis* cells that bore a septum. Two independent experiments were quantified. (C) Super-resolution photographs of *M. smegmatis* cells untreated or treated with 0.5×, 2×, and 10× MIC of meropenem for 4 h. Scale bar, 2 μm. Green arrowhead, open septum; blue arrowhead, closed septum; red arrowhead, clubbed pole. (D) High-resolution photographs of *M. tuberculosis* cells untreated or treated with 0.5× and 2× MIC of meropenem for 24 h. Scale bar, 2 μm. Blue arrowhead, a shorter cell with one swollen pole; yellow arrowhead, a longer dividing cell with two swollen poles. (E) Super-resolution photographs of *M. tuberculosis* cells treated with 0.5× MIC of meropenem for 24 h. Scale bar, 2 μm.

As an example, we treated *M. smegmatis* and *M. tuberculosis* with sublethal or bactericidal concentrations of meropenem for 4 and 24 h, respectively. For both species, FDAA incorporation decreased in proportion to the meropenem concentration and meropenem caused distinct morphometric abnormalities (Fig. 5A and C to E). In meropenem-treated *M. smegmatis* cells, the FDAA signal was greater at the subpolar region than in the rest of the cell, except for the septum (Fig. 5A and C). One pole was affected more than the other, producing polar clubbing, and most cells accumulated FDAAs in the subpolar region of the pole that was clubbed (Fig. 5A and C). Moreover, the proportion of *M. smegmatis* cells that bore a septum increased dramatically upon meropenem treatment (Fig. 5A and B). Most septa appeared as two foci rather than a segment that oriented perpendicularly to the longitudinal axis of a cell, suggesting that septation was incomplete (Fig. 5A). This was confirmed by 3-D–SIM (Fig. 5C). This has prompted us to speculate that two separate machineries initiate the formation of a septum and ensure its closure, as has been reported in *Streptococcus pneumoniae* strain D29 cells (47), and that meropenem inhibits the machinery responsible for septal closure. Visual inspection of meropenem-treated *M. tuberculosis* cells suggested that morphological alterations varied: one pole appeared chiefly altered in some cells, and the two poles were more similarly swollen in others. Moreover, FDAAs were well incorporated at the malformed pole(s) (Fig. 5D and E).

The spatiotemporal analysis of FDAA incorporation described here raises new questions about the enzymology, topology, and temporal regulation of PG synthesis in mycobacteria, particularly about the capacity for PG synthesis to mature in the new pole of mycobacterial cells after the separation of a progeny pair. Answers to these newly framed questions may lead to the identification of new drug targets and better understanding of the mechanisms of action of existing antimycobacterial drugs.

## MATERIALS AND METHODS

### Construction of *M. smegmatis* cells expressing Wag31-mCherry.

The gene *wag31* was amplified from the genomic DNA of *M. tuberculosis* using the following primer pair: attB2_SD_wag31, GGGGACAGCTTTCTTGTACAAAGTGGCAGAAAGGAGGTTAATAATGCCGCTTACACCTGCCGACGTCCAC, and Linker Wag31, ATGCCCGAGCCCGAGCCCGAGTTTTTGCCCCGGTTGAATTGATCGAAGCCACC. The reporter construct mCherry was fused to the C terminus of Wag31. The nucleotide sequence TCGGGCTCGGGCTCGGGC encoding the protein linker SGSGSG was introduced between the nucleotide sequences encoding the protein of interest and the reporter. The plasmid pGMCZ-T0X-PTB38 *wag31*-*mCherry* allowed integration of Wag31-mCherry into the chromosome and constitutive expression under the control of the strong promoter TB38.

### Bacterial strains and culture conditions.

*M. smegmatis*, *M. smegmatis* Wag31-mCherry, and *M. tuberculosis* were cultured at 37°C in Middlebrook 7H9 medium (BD Difco) supplemented with 0.2% glycerol, 0.5% bovine serum albumin fraction V, 0.05% Tween 80, 0.2% dextrose, 0.085% NaCl, and 0.02% tyloxapol. Zeocin was added at a final concentration of 25 μg/ml when applicable. Cultures were grown in 50-ml Falcon tubes at a 1:100 liquid/air ratio.

### Synthesis of FDAAs and FLAA.

All fluorescent d-alanine analogs used in this study were synthesized following the procedure described by Kuru and colleagues (48) and were purified using a Waters Acquity ultraperformance liquid chromatography (UPLC) system equipped with a 1.7-μm Acquity UPLC BEH C_18_ column (1.7-μm particle size, 2.1 by 100 mm) using a 10-to-30% gradient of acetonitrile-water containing 0.01% trifluoroacetic acid (TFA) over a 5-min run, including a 3-min wash. The areas under the peaks were calculated using MassLynx software (Waters, Inc.). The degrees of purity of HADA, NADA, and HALA were >97%, >97%, and >98%, respectively.

### Treatment of mycobacterial cells with meropenem.

*M. tuberculosis* and *M. smegmatis* cultures were inoculated at an optical density at 600 nm (OD_600_) of 0.1 into 7H9 medium supplemented with 1 mM HADA. Meropenem was freshly resuspended in dimethyl sulfoxide (DMSO) and was further added at 0.5 µg/ml (0.5× MIC) or 2 µg/ml (2× MIC) for *M. tuberculosis* and 4 µg/ml (0.5× MIC), 16 µg/ml (2× MIC), or 80 µg/ml (10× MIC) for *M. smegmatis*. For *M. tuberculosis* experiments, clavulanate was added at 10 µM. Cultures were perfused with meropenem at 37°C for 4 h and 24 h for *M. smegmatis* and *M. tuberculosis*, respectively.

### Heat inactivation.

Cells were heat inactivated at 90°C for 15 min prior to a 4-h incubation in 7H9 medium supplemented with HALA or HADA. No CFU were recovered after heat treatment.

### High-resolution microscopy.

For imaging, bacterial suspensions were deposited on soft agar pads and visualized using a DeltaVision image restoration microscope (GE Healthcare) equipped with an Olympus IX-71 microscope with a 100×/1.4 numeric aperture (NA) UPlanSApo objective and appropriate filter sets (for DAPI, excitation at 390/10 and emission at 435/48, and for fluorescein isothiocyanate [FITC], excitation at 475/28 and emission at 525/48), a pco.edge scientific complementary metal oxide-semiconductor (sCMOS) camera (PCO-Tech), and an Insight SSI 7-color solid-state illumination system.

### 3-D–SIM super-resolution microscopy.

For super-resolution imaging, bacterial suspensions were mixed with the same volume of mounting medium (ProLong gold antifade mountant; Thermo Fisher Scientific), and 10-μl amounts were spread on microscope slides (75- by 25-mm Corning 2948) and covered with high-precision microscope cover glasses (1.5H; GmbH). Super-resolution microscopy data were acquired using a DeltaVision OMX V4/Blaze 3-D–SIM super-resolution microscope (GE Healthcare) housed in the Rockefeller University Bio-Imaging Resource Center (BIRC). This OMX system is fitted with a 100×/1.40 UPLSAPO oil objective (Olympus), Evolve electron-multiplying charge-coupled device (EMCCD) cameras (Photometrics) used in EM gain mode at a gain of 170, 405-nm and 488-nm excitation lasers, and 436/31 and 528/48 emission filters. Optical sections were acquired at 125-nm intervals. The immersion oil refractive index was selected to optimize for the 488 channel and the ambient temperature. Structured illumination data sets were reconstructed using softWoRx software (GE Healthcare), employing optical transfer functions (OTFs) generated from point spread functions acquired from subresolution beads, channel-specific k0 (stripe rotation angle) values, and a Wiener filter of 0.002. The image registration parameters and OTFs were refined by the BIRC staff.

### PG labeling using FDAAs.

*M. tuberculosis* and *M. smegmatis* cultures were inoculated at an OD_600_ of 0.1 to 0.3 into 7H9 medium supplemented with 1 mM HADA or 1 mM NADA. When sequential labeling was performed, bacterial suspensions perfused with the first FDAA were washed 3 times with 7H9 medium and further incubated in 7H9 medium supplemented with the second FDAA. For both single and sequential labeling, bacterial suspensions were washed 3 times with PBS–0.05% Tween 80 and fixed with 4% paraformaldehyde for 30 min and 4 h for *M. smegmatis* and *M. tuberculosis*, respectively, to ensure bacterial death for further imaging outside a contained environment. Of note, incubation with a first FDAA prior to the incubation with a second one helped us assess whether cells grew normally; most did in growth-permissive, rich medium. As such, the first label was not strictly required and did not serve the analysis detailed below.

### Image analysis and statistical analysis.

We evaluated the incorporation of FDAAs into cells that did not bear a septum; cells that had a septum were excluded from the analysis. As such, this study reports the FDAA incorporation of cells spanning the cell cycle period that corresponds to 0.17 (0.5/3) cycles prior to cell separation to septum formation. Therefore, a minority of cells that have been included in the analysis may have just separated. Images were processed with the open-source program ImageJ (http://imagej.nih.gov/ij/) or Icy (http://icy.bioimageanalysis.org/) (49). Data were compiled using MatLab (MathWorks). Briefly, using ImageJ, we drew a segmented line that followed the longitudinal axis of a cell and measured the fluorescence intensities along it. Next, we used customized MatLab algorithms to automatically compute the logarithm of the ratio of the total fluorescence of the two halves of a cell. All cells were processed similarly. The statistical analysis was performed using MatLab.
